# Continuous Vital Sign Analysis to Predict Secondary Neurological Decline After Traumatic Brain Injury

**DOI:** 10.3389/fneur.2018.00761

**Published:** 2018-09-25

**Authors:** Christopher Melinosky, Shiming Yang, Peter Hu, HsiaoChi Li, Catriona H. T. Miller, Imad Khan, Colin Mackenzie, Wan-Tsu Chang, Gunjan Parikh, Deborah Stein, Neeraj Badjatia

**Affiliations:** ^1^Program in Trauma, University of Maryland School of Medicine, Baltimore, MD, United States; ^2^Department of Neurology, University of Maryland School of Medicine, Baltimore, MD, United States; ^3^Department of Anesthesiology, University of Maryland School of Medicine, Baltimore, MD, United States; ^4^Enroute care Division, Department of Aeromedical Research, U.S. Air Force School of Aerospace Medicine, Wright Patterson AFB, Dayton, OH, United States; ^5^Department of Emergency Medicine, University of Maryland School of Medicine, Baltimore, MD, United States; ^6^Department of Surgery, University of Maryland School of Medicine, Baltimore, MD, United States

**Keywords:** traumatic brain injury, machine learning, heart rate variability, photoplethysmogram, predictive model

## Abstract

**Background:** In the acute resuscitation period after traumatic brain injury (TBI), one of the goals is to identify those at risk for secondary neurological decline (ND), represented by a constellation of clinical signs that can be identified as objective events related to secondary brain injury and independently impact outcome. We investigated whether continuous vital sign variability and waveform analysis of the electrocardiogram (ECG) or photoplethysmogram (PPG) within the first hour of resuscitation may enhance the ability to predict ND in the initial 48 hours after traumatic brain injury (TBI).

**Methods:** Retrospective analysis of ND in TBI patients enrolled in the prospective Oximetry and Noninvasive Predictors Of Intervention Need after Trauma (ONPOINT) study. ND was defined as any of the following occurring in the first 48 h: new asymmetric pupillary dilatation (>2 mm), 2 point GCS decline, interval worsening of CT scan as assessed by the Marshall score, or intervention for cerebral edema. Beat-to-beat variation of ECG or PPG, as well as waveform features during the first 15 and 60 min after arrival in the TRU were analyzed to determine physiologic parameters associated with future ND. Physiologic and admission clinical variables were combined in multivariable logistic regression models predicting ND and inpatient mortality.

**Results:** There were 33 (17%) patients with ND among 191 patients (mean age 43 years old, GCS 13, ISS 12, 69% men) who met study criteria. ND was associated with ICU admission (*P* < 0.001) and inpatient mortality (*P* < 0.001). Both ECG (AUROC: 0.84, 95% CI: 0.76,0.93) and PPG (AUROC: 0.87, 95% CI: 0.80, 0.93) analyses during the first 15 min of resuscitation demonstrated a greater ability to predict ND then clinical characteristics alone (AUROC: 0.69, 95% CI: 0.59, 0.8). Age (*P* = 0.02), Marshall score (*P* = 0.001), penetrating injury (*P* = 0.02), and predictive probability for ND by PPG analysis at 15 min (*P* = 0.03) were independently associated with inpatient mortality.

**Conclusions:** Analysis of variability and ECG or PPG waveform in the first minutes of resuscitation may represent a non-invasive early marker of future ND.

## Introduction

One and a half million Americans incur a traumatic brain injury (TBI) each year (1) and approximately 5.3 million individuals have enduring disabilities as a direct result of a TBI (1). In the acute resuscitation period, one of the goals is to identify those at risk for secondary neurological decline, represented by a constellation of clinical signs that can be identified as objective events related to secondary brain injury and independently impact outcome (2). In moderate to severe TBI, the incidence of secondary neurological decline is approximately 20% (3), and the negative impact of many of these events, e.g., rise in intracranial pressure or need for decompressive craniectomy, can be potentially minimized if identified early. In mild TBI populations, while delayed neurological decline is a relatively rare event [estimated to be 5–10%(4)], patients often require a lengthy observation and stays to rule out the possibility of secondary decline. Currently, initial risk stratification after injury is based on clinical judgment, clinical examination, and assessment of static vital signs (5), which only provide partial guidance in the acute resuscitation period.

Additional essential information can be derived from the analysis of continuous vital sign data and waveform analysis. Specifically, autonomic nervous system (ANS) function can be monitored non-invasively and continuously by analyzing variability and waveform features from routine electrocardiogram (ECG) or photoplethysmogram (PPG) monitoring (6–8).

We hypothesized that in combination with routine clinical assessments and neuroimaging in the first hour after injury, continuous vital sign monitoring for variability and waveform feature analyses from either the ECG or PPG can be utilized to reliably predict early (<48 h after injury) ND in a cohort patients with isolated TBI patients.

## Methods

### Patient selection and study design

This is a retrospective subgroup analysis using data collected for the project Oximetry and Noninvasive Predictors Of Intervention Need after Trauma (ONPOINT) project (FA8650-11-2-6D01) at the University of Maryland Medical Center, R Adams Cowley Shock Trauma Center Trauma Resuscitation Unit (TRU). ONPOINT was a project designed to identify new noninvasive sensors and physiological features that may predict trauma patients' need for lifesaving interventions (9–11). Patients were included in ONPOINT if they had a shock index of 0.62 or greater based on VS radioed in from the field by the emergency medical service (EMS) provider, were designated EMS “Priority 1” (denoting a critically ill or injured person requiring immediate attention), or were unstable with a life-threatening injury but without an available prehospital VS. Patients who survived less than 15 min after admission to the trauma center were excluded, as were patients with neurological impairment due to cervical spine injury. In addition, this subset analysis was limited to patients with a head abbreviated injury score (AIS)>1 who had at least 2 CT scans done within the first 24 h and availability of PPG and ECG waveform data for the first hour of admission. Patients with significant systemic trauma, identified as abdominal or thoracic AIS>1 were excluded.

Charts were retrospectively reviewed for ND from the time of arrival in the TRU through the first 48 h of admission. Clinical data were gathered from recorded hourly bedside neurologic assessments of GCS and pupillary reactivity and neurosurgical notes for surgical intervention. Radiographic data was gathered from Marshall scores of the first three CT scans done in the first 24 h. Demographic and injury severity data were obtained from the institutional Trauma Registry. Continuous PPG and ECG 240 Hz waveforms were collected via BedMaster® (Excel Medical Electronics Inc., Jupiter, FL) vital signs collection system beginning at the time of arrival in the TRU.

### Data processing and feature design

Signal quality was evaluated based on R-peaks in ECG and the peaks in PPG, with the assumption that good quality signals have normal distributed R-R intervals. R-R intervals from segments of low quality were detected as outliers using the Z-test. An additional Figure shows this more detail (Supplemental Figure 1).

**Figure 1:**
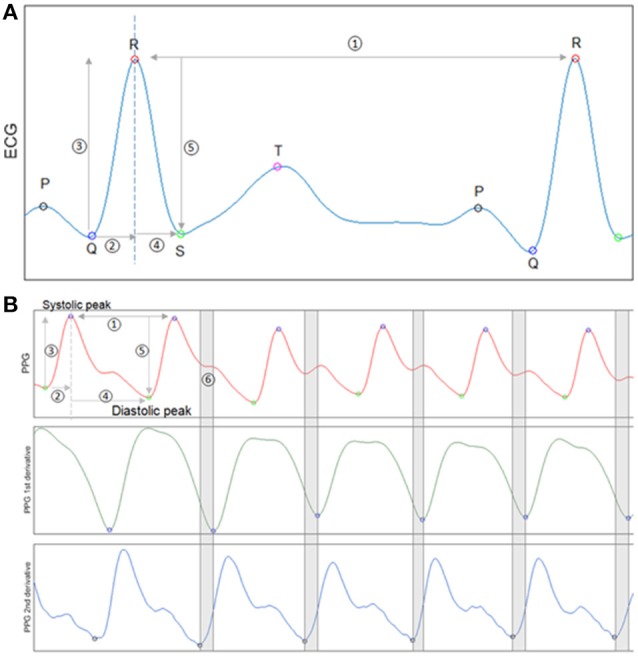
Waveform feature analysis for ECG and PPG. **(A)** An exemplary ECG segment with identified P,Q,R,S,T peaks. Five items from the segment are used for ECG feature calculation. Item 1 is the NN interval. Items 2 and 4 are Q to R rising time and R to S falling time. Items 3 and 5 are Q to R rising amplitude and R to S falling amplitude. **(B)** An exemplary PPG segment with identified peaks and valleys (1st panel).The first derivative of PPG signal (2nd panel). The second derivative of PPG signal (3rd panel). The first and second derivatives of the PPG waveform were used to identify the diastolic notch indicated by segment 6.

HRV and waveform morphology features were derived from the ECG. Figure 1 shows a typical PQRST segment from ECG, with identified peaks and five items that were used for calculation. The normal-to-normal (NN) interval illustrated by item 1 is the time interval between two consecutive R peaks. HRV was measured beat to beat variability using standard definitions. HRV variables in time domain and nonlinear dynamics were calculated based on the Task Force of the European Society of Cardiology and the North American Society of Pacing and Electrophysiology (12, 13). From items 2 and 4 in Figure 1, the rising time from Q to R and the falling time from R to S were calculated. Similarly, from items 3 and 5, the rising and falling amplitudes from Q to R and R to S were calculated. Statistical quantities, such as the 1st, 2nd, 3rd quartiles, minimum, maximum, and Shannon entropy were used to summarize each above variable in a selected time window.

PPG variability and waveform morphology features were also designed similarly with expansion based on PPG unique characteristics. Figure 1B subplot shows a normal PPG segment with identified peaks and valleys. Item 1 illustrates a peak-to-peak time interval, which is analog to the NN interval in ECG. PPGV variables and morphology features were calculated from items 1–4 as we did for ECG. PPG waveform also has unique dicrotic notch that its shape has been studied and shown to be related to arterial stiffness and aging (14). To measure the deceleration and acceleration near the dicrotic notch, the first and second derivatives of PPG were calculated through three-point central difference (15, 16).

We only utilized continuous vital sign data from the initial 15 and 60 min of arrival to the TRU to develop PPG and ECG derived models. The waveform and physiologic variables analyzed for both PPG and ECG are shown in Supplemental Table 1. Multiple models were created for analysis (Table 1). The first set of models utilized clinical characteristics typically available within the first hour of patient arrival. The second set of models utilized only physiologic data from the first 15 and 60 min after arrival in the TRU. The final set of models combined clinical characteristics with ECG and PPG analyses at 60 min after arrival to the TRU.

**Table 1:** Characteristics of models to predict neurological worsening.

**Model**	**Variables**
**CLINICAL CHARACTERISTICS**	
Model 1	Age, sex, first vital sign data recorded (HR, RR, SBP, DBP)
Model 2	Age, sex, first vital sign data recorded (HR, RR, SBP, DBP), and initial GCS recorded
Model 3	Age, sex, first vital sign data recorded (HR, RR, SBP, DBP), initial GCS, and Marshall score
**PHYSIOLOGIC CHARACTERISTICS**	
Model 4	ECG heart rate variability and waveform feature analysis for the first 15 min
Model 5	PPG variability and waveform feature analysis for the first 15 min
Model 6	ECG heart rate variability and waveform feature analysis for the first 60 min
Model 7	PPG variability and waveform feature analysis for the first 60 min
**COMBINED CLINICAL AND PHYSIOLOGIC CHARACTERISTICS**	
Model 8	Model 2 + Model 4
Model 9	Model 2 + Model 5

### Outcome measures

#### Neurological decline (ND)

ND was defined using previously reported criteria as any of the following occurring in the first 48 h: new asymmetric pupillary dilatation(>2 mm), 2 point GCS decline not due to intubation for procedure, or analgo-sedation; interval worsening of CT scan as assessed by the Marshall score (17); treatment of cerebral edema by placement of intracranial monitoring (intracranial parenchymal pressure monitor or external ventricular drainage), or treatment by osmotherapy, hyperventilation, craniotomy or decompressive craniectomy. Two investigators (NB, GP) independently reviewed at least 2 CT scans during study period for each patient and provided a Marshall score for each scan. Changes in GCS and pupillary size were determined by assessing the hourly score and pupil size recorded in the nursing flowsheet. Influence of analgo-sedation was determined by reviewing medical records and eliminating any decline in exam that occurred within 2 h of medication administration. Decline not related to radiographic worsening was labeled as clinical ND, whereas interval worsening Marshall score was labeled as radiographic ND. Mortality within 48 h was abstracted from the chart.

### Statistical analysis

Neurological decline was classified either present or absent, if any of the a priori criteria for ND were met. Multivariate stepwise logistic regression was used to establish the association between clinical, ECG, and PPG variables and neurological decline. The Wald Chi-square test was used to determine whether one variable should be included (forward step) or excluded (backward step). To test models' generalization capability on new data, 10-fold cross-validation repeated 10 times with stratified sampling was used (18). Area under the receiver operating characteristic curve (AUROC) was used as an overall performance metric. Lack of overlap in 95% confidence interval (CI) was considered statistically significant. Sensitivity, specificity, positive predicted value (PPV) and negative predicted value (NPV) were calculated from the optimal threshold based on the Youden index. All signal processing and feature extracting were analyzed with Matlab (2014b, Mathworks, Natick, MA). Multivariable models were built to determine whether the prediction probability of PPGV for ND was independently associated with in patient mortality.

Predictive models and statistical analysis were implemented with R software version 3.2.2 (R Development Core Team, Vienna, Austria).

Approval for this study was obtained from the University of Maryland School of Medicine and US Air Force Research Laboratory Institutional Review Boards.

## Results

**Figure 2:**
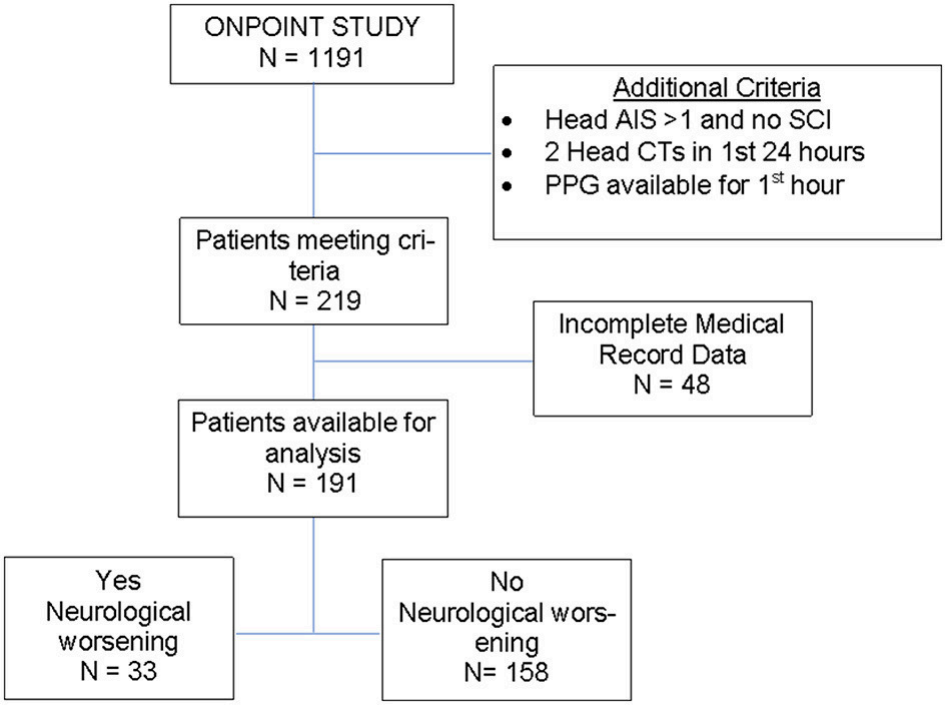
Flow diagram of study cohort.

There were 1191 patients admitted satisfying the age (≥18 years old) and pre-hospital SI (≥ 0.62) criteria for inclusion in the ONPOINT study between December 2011 and May 2013. There were 219 cases that met additional criteria for this subgroup analysis, and of those, 191 cases were found to have complete data for analysis (Figure 2). ND was found to have occurred in 17% of patients (*n* = 33/191) with baseline characteristics shown in Table 2. Clinical ND occurred in 19 (10%), radiographic worsening in 25 (13%). In the 11 (6%) patients that had both clinical and radiographic worsening only 1 ND event was counted for the final analysis. ND was associated with a longer hospital length of stay(*P* < 0.001), admission to the intensive care unit (*P* < 0.001) and higher rates of in patient mortality (*P* < 0.001). These data are shown in more detail in Supplemental Table 2.

**Table 2:** Baseline characteristics of study cohort.

**Admission characteristics**	**Neurological decline**		
	**No**	**Yes**	
	***N* = 158**	***N* = 33**	***P*-value**
Age	41 (25, 54)	48 (26, 74)	0.19
Men	112 (71)	25 (76)	0.61
RACE			0.64
White	103(65)	23 (70)	
Black	40 (25)	10 (30)	
Other	15 (10)	–	
Injury severity score	5 (5, 14)	25 (16,29)	<0.001
GCS score	15 (15, 15)	7 (3, 14)	<0.001
Marshall score	1 (1, 1)	2 (1, 3)	<0.001
MAP(mmHg)	107 (98,118)	108 (102, 118)	<0.001
Heart Rate (bpm)	97 (82,106)	88 (75, 103)	0.39
Resp. Rate (bpm)	20 (17, 23)	18 (12, 24)	0.08
*O*_2_ Saturation (%)	99 (98, 100)	100 (97, 100)	0.45
Mechanism of injury			0.21
Blunt	147 (93)	28 (85)	
Penetrating	11 (7)	5 (15)	

*All continuous data shown at median (25th%ile, 75%thile). Categorical data shown as n(%). P-values obtained from Chi-Square test and Mann-Whitney U test for categorical and continuous data respectively*.

### ND predictive model development and performance (Table 3)

#### Clinical characteristics

A model accounting for the initial VS measurements, age, and sex (Model 1) was the weakest model but able to predict ND within the next 48 h (AUC 0.69, 95% CI:0.59, 0.8 *P* = 0.0002); adding admission GCS to the model resulted in improvement (AUC 0.86, 95% CI: 0.77, 0.94, *p* < 0.0001). Incorporating Marshall scores to admission data further enhanced predictability (AUC 0.90, *p* < 0.0001).

#### Heart rate variability and waveform feature analysis

ECG HRV and waveform feature analyses during the first 15 (model 4) and 60 min (model 6) post admission to the TRU demonstrated a strong ability to predict ND. Similarly, PPG variability and waveform feature analysis has a high AUROC at both 15 (model 5) and 60 (model 7) min for predicting ND. Both sets of models had high negative predictive values (NPV).

#### Combined models of clinical characteristics and physiologic data

Combining clinical characteristics, including GCS, with physiologic data obtained from ECG (model 8) or PPG (model 9) yielded similar AUROC results, with a strong NPV of 0.97 and 0.98, respectively.

### Prediction of in—hospital mortality

The predictive probabilistic score of PPG feature analysis at 15 min for ND (PPG15) was entered into a multivariable logistic regression model determining factors associated with inpatient mortality. Older age (Odds Ratio (OR): 1.05, 95% CI: 1.01, 1.09, *P* = 0.02), Marshall CT score (OR: 4.2, 95% CI: 18.6, 9.47, *P* = 0.002), penetrating injury (OR: 2.9, 95% CI: 1.16, 7.22, *P* = 0.02), and PPG15 (OR: 3.26, 95% CI: 1.14, 9.4, *P* = 0.03) were found to be independently associated with a higher risk of inpatient mortality, after adjusting for sex, admission mean arterial pressure (MAP), heart rate, respiratory rate, and GCS.

## Discussion

Without regards to the patient age, sex, GCS or initial set of vital signs, the PPG and ECG analyses alone at 15 min (models 4 and 5) were better or as good as clinical models 1 and 2 (Table 3) to predict ND. Moreover, a combined model with the clinical and physiologic characteristics at 15 and 60 min after arrival yielded an improved prediction power for ND. These analyses reflect the great potential assessing the physiological state by utilizing continuous vital sign data to enhance the clinical assessment in the acute resuscitation period after TBI.

**Table 3:** Predictive models of neurological decline.

**Model**	**AUROC^a^**	**AUROC 95% CI**		**PPV^b^**	**NPV^c^**
		**Low**	**High**		
**CLINICAL CHARACTERISTICS**					
Model 1	0.69	0.59	0.80	0.35	0.90
Model 2	0.86	0.77	0.94	0.67	0.94
Model 3	0.90	0.84	0.97	0.61	0.97
**PHYSIOLOGIC CHARACTERISTICS**					
Model 4	0.84	0.76	0.93	0.53	0.94
Model 5	0.87	0.80	0.93	0.47	0.95
Model 6	0.89	0.83	0.96	0.57	0.96
Model 7	0.83	0.74	0.91	0.47	0.94
**COMBINED CLINICAL AND PHYSIOLOGIC CHARACTERISTICS**					
Model 8	0.92	0.87	0.97	0.76	0.97
Model 9	0.92	0.86	0.98	0.68	0.98

a *AUROC, Area Under the Curve for Receiver Operator Curve*.

b *PPV, positive predictive value*.

c *NPV, negative predictive value*.

ECG and PPG sensors provide a wealth of information on the cardiovascular and respiratory systems through further waveform analysis. The ECG waveform can provide data on the heart rate variability (HRV) which has been used in studies of neurologic disorders (13, 19) as a marker of the function of the ANS (20) Specific to TBI, dysautonomia has been closely linked to increased ICP or decreased CPP (21). Baguley et al. (22) observed that in TBI, patients with and without dysautonomia showed HRV differences compared to controls. The pulse oximeter is a commonly used sensor that can provide rich data by generation of a PPG waveform, which can provide information on heart rate, oxygen saturation, and respiratory rate (23). The PPG peaks correspond to the R peaks from ECG, therefore, the peak-peak interval from PPG can be used as an alternative to the NN interval calculated from ECG recordings. Lu et al. (24) found PPG variability (PPGV) was highly correlated to HRV and could serve as an alternative measurement. Several studies have correlated similar physiological measurements with autonomic changes in TBI patients with the severity of injury and, association with increased ICP (25–27) as well as overall morbidity and mortality (25, 27, 28).

Our results are novel given we only utilized continuous ECG and PPG data from the first minutes of arrival, prior to ICP monitoring and CT imaging, further signifying the viability of physiologic and waveform analysis as a robust early marker in the resuscitation phase. It is important to note that within 15 min of arrival, patients were still undergoing physical exam and assessment, and were not under the influence of sedatives or analgesics that may have confounded the signal from the continuous vital sign data collection. The ability to accurately discern a risk for ND early in the acute resuscitation phase could provide for an opportunity to develop targeted interventions to mitigate secondary injury, including rapid triage for timely, definitive treatment.

The results from this study also indicate that ND within the first 48 h after injury occurs commonly after isolated TBI, and that it is associated with a longer duration of hospitalization and higher rate of mortality. A recent analysis of mild TBI patients found a mortality rate of 23% in patients who had acute ND (4). Our study had a higher rate of inpatient mortality likely due to a higher baseline severity of injury.

ND prediction with admission VS, age, sex and GCS score was moderately accurate, confirming the importance of a thorough clinical assessment upon arrival to the hospital. GCS is a robust neurological assessment tool; however, early presentation can be deceiving, with many low energy falls resulting in brain hemorrhage and swelling, resulting in increased ICP and greater risk for severe disability and death (29–33). Additionally, the inter-rater reliability of GCS measurement is variable, and may be influenced by level of training and experience of the individual making the assessment (34–36). This further highlights the importance of utilizing metrics, such as ECG or PPG waveform feature analysis, that are resilient to factors that impact the reliability of the GCS assessment. Serial CT imaging provides additional information regarding clinically silent progression of injury (37–39) with the aim of capturing signs of neurologic worsening which can lead to early medical and surgical interventions even before the clinical symptoms manifest (40). Despite our results indicating additional benefit of scoring severity of injury by CT imaging, a multicenter prospective study as well as several large single center cohort studies have failed to find factors that reliably predicted the correlation between ND and serial CT imaging (41–44). Serial CT imaging is likely most useful as a practice for patients in whom examination is not reliable and/or as a confirmatory test for assessing structural reasons for ND. Moreover, serial imaging is often not practical in the rural areas, developing countries and combat fields with limited resources.

There are limitations to this study that are worth considering. This study was a retrospective analysis in which there may be reporting bias for ND. Due to this concern, we measured variables that we know are entered with high reliability, such as GCS score, change in pupil size, and radiographic changes. The timing of the imaging was not always consistent. However, most TBI patients at our institution receive a head CT within the hour of arrival. During CT, vital sign data is not able to be collected, however, our continuous data capture system was able to capture the majority of waveform data during the study period. Though we conducted a rigorous testing process by 10-fold cross-validation repeated 10 times with stratified sampling, the results here are from a single center and need validation in a prospective cohort. This was a population without polytrauma and a high initial GCS scores indicating mild TBI. We intentionally chose a population of relatively mild TBI without polytrauma in order to test our hypotheses; a broader population of TBI patients with polytrauma need to be tested in order to increase the generalizability of our findings. Sedation and analgesia may have played a role in exam features, though exam changes occurring following sedation was not labeled ND.

## Conclusions

By coupling patient factors and continuous physiologic data, a non—invasive predictive model of neurological deterioration is feasible and may potentially lead to automated decision support algorithms and provide for earlier, targeted therapeutic interventions. This may be especially useful in rural or austere settings where acute triage decisions may be made without the benefit of neuroimaging or subspecialty expertise. Further refinement in larger, more heterogeneous groups of TBI patients is necessary prior applying these findings in a prospective setting.

## Author contributions

NB, ChM, SY, PH, and CaM made substantial contributions to conception and design, analysis and interpretation of data. GP, WC, HL, ChM, and IK made substantial contributions to the acquisition of data, analysis and interpretation of data. NB, ChM, SY, PH, GP, and DS were involved in drafting the manuscript or revising it critically for important intellectual content. All authors given final approval of the version to be published. All authors agreed to be accountable for all aspects of the work in ensuring that questions related to the accuracy or integrity of any part of the work are appropriately investigated and resolved.

### Conflict of interest statement

The authors declare that the research was conducted in the absence of any commercial or financial relationships that could be construed as a potential conflict of interest. The reviewer SS and the handling Editor declared their shared affiliation at the time of the review.
