# Supplementary material for: Continuous Vital Sign Analysis to Predict Secondary Neurological Decline After Traumatic Brain Injury
Source: Front Neurol. 2018 Sep 25;9:761. doi: 10.3389/fneur.2018.00761 (PMC6167472; doi:10.3389/fneur.2018.00761)
Supplement: Supplementary file 1 [file Table_1.DOCX]

| **Variable** | **Description** |
| --- | --- |
| ECG waveform | square root of the mean squared differences of successive NN intervals |
|  | Coefficient of variation of NN intervals |
|  | Poincare plot analysis SD2 |
|  | Detrended fluctuation analysis alpha2 |
|  | Irreversibility analysis as1 |
|  | Maximum Q to R rising amplitude |
|  | Medium Q to R rising amplitude |
|  | 3^rd^ quartile Q to R rising amplitude |
|  | Minimum R to S falling time |
|  | Maximum R to S falling time |
|  | Minimum R to S falling amplitude |
|  | 1^st^ quartile R to S falling amplitude |
|  | Medium R to S falling amplitude |
| PPG | square root of the mean squared differences of successive NN intervals |
|  | Coefficient of variation of NN intervals |
|  | Percentage of NN intervals differences < 20 ms |
|  | Detrended fluctuation analysis alpha2 |
|  | Irreversibility analysis as1 |
|  | 3^rd^ quartile Systolic rising time |
|  | 3^rd^ quartile Diastolic falling time |
|  | Minimum Systolic and diastolic period ratio |
|  | 3^rd^ quartile Systolic and diastolic period ratio |

Supplemental Table 1. Physiologic and Waveform Data Selected in Multivariable Analyses
