# Supplementary material for: Continuous Vital Sign Analysis to Predict Secondary Neurological Decline After Traumatic Brain Injury
Source: Front Neurol. 2018 Sep 25;9:761. doi: 10.3389/fneur.2018.00761 (PMC6167472; doi:10.3389/fneur.2018.00761)
Supplement: Supplementary file 2 [file Table_2.DOCX]

| **Supplemental Table 2. Outcomes associated with Neurological worsening Event** | | | |
| --- | --- | --- | --- |
|  |  |  |  |
|  | Neurological Worsening | |  |
|  | No (N=158) | Yes (N=33) | P value |
| TRU*^a^* Disposition |  |  | <0.001 |
| Home | 99 (63) | 4 (12) |  |
| Intensive Care Unit | 10 (6) | 23 (70) |  |
| Intermediate Care Unit | 31(20) | 6 (18) |  |
| General Trauma Ward | 18 (11) | -- |  |
| Mortality | 1 (1) | 14 (42) | <0.001 |
| Length of Stay (days) | 0.5 (0.2,2.3) | 3.6 (1.3,9.4) | <0.001 |
| *^a^*TRU - Trauma Resuscitation Unit. All continuous data shown at median (25th%ile, 75th%ile). Categorical data shown as n(%). P values obtained from Chi-Square test and Mann-Whitney U test for categorical and continuous data respectively. | | | |
