# Supplementary figures and images for: Continuous Vital Sign Analysis to Predict Secondary Neurological Decline After Traumatic Brain Injury
Source: Front Neurol. 2018 Sep 25;9:761. doi: 10.3389/fneur.2018.00761 (PMC6167472; doi:10.3389/fneur.2018.00761)

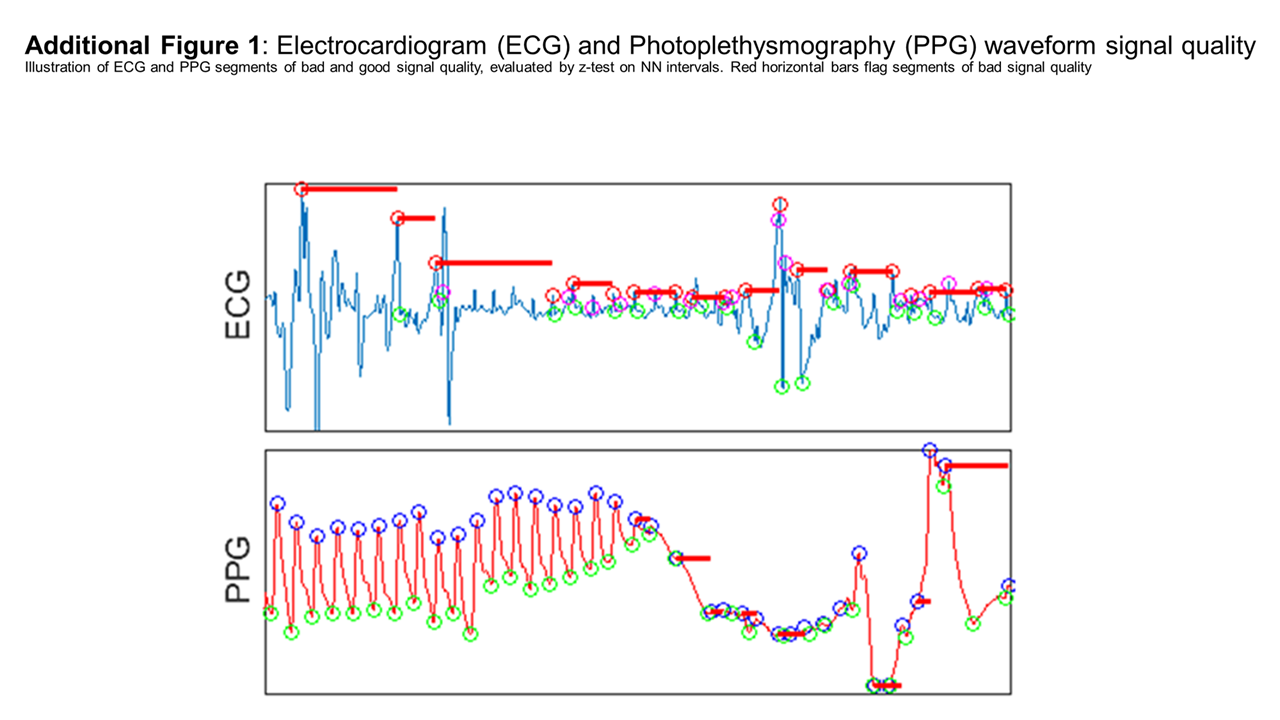

Supplement: Supplementary file 3 [file Image_1.TIF]
